# What Is a Disease: What Is the Meaning of Diagnosis?

**Published:** 1907-10-12

**Authors:** Sydney W. Macilwaine

**Affiliations:** (retired), Peterhurst, Cleveden, Somerset.


### SPECIAL ARTICLE.

### WHAT IS DISEASE? WHAT IS DIAGNOSIS?

By SYDNEY W. MACILWAINE, M.R.C.S., L.R.C.P. (retired), Peterhurst, Cleveden, Somerset.

To most medical men it may seem at first sight unnecessary to attempt to answer the question at the head of this paper; for some will think they have a very definite idea of the meaning of a disease and its diagnosis, while others will recall what their teachers and text-books taught them, namely, that it is impossible to attach a definite meaning to the specific word disease. I would urge them all to rid their minds for the moment of preconceived ideas. It is also necessary to remember that medicine consists of two distinct and indeed separable parts, a pure science and its application, the art of healing. In attempting to answer this question we are concerned wholly and solely with pure science.

The word disease has two meanings, and one of these need only be mentioned to be got rid of. In the general, negative sense disease is simply the negation of health, health being taken to represent the state accompanying normal life. This use of the word corresponds exactly with the use of the word cold as the negation of heat, and it does not concern us further at present. The question here raised applies to the word disease used in the particular, positive sense.

Typhoid fever, lead-poisoning, and heat-stroke are diseases, as no one will dispute, and they represent three very large groups of diseases. Typhoid fever is due to the parasitism of a well-known microbe; lead-poisoning is due to the ingestion of a poison; heat-stroke is due to exposure to hurtful physical conditions. If account be taken of every form of trauma from excessive heat to the ordinary kinds of violence, if it be remembered how many diseases are due to parasitism, and how many to the ingestion of poisonous or unwholesome matter—including excessive or deficient diet—it will be seen that the vast majority of the diseases at present recognised are included in these groups. Reflection will show that every case of every one of this great array of diseases agrees with every other in this, that it consists of cause and effect. In other words, every attack of every one of these diseases is a reaction between man and his environment; it is a natural process.

It is therefore clear beyond possibility of misunderstanding what is meant by, for instance, "the disease typhoid fever." It is a conception drawn from the observation of a vast series of similar natural processes. Our conception of typhoid fever is no less definite nowadays than our conception of any other natural process, such as oxidation. It must surely be clear to all that the stability of the fabric of medicine is dependent on the definiteness of our conceptions of the specific diseases, for these conceptions are the only standard by which we can gauge the correctness of our diagnosis in any case of sickness submitted to us. So soon as we reach a definite meaning for the specific word disease we fix the value of diagnosis; every disease represents cause and effect; therefore diagnosis means the tracing of effect to cause in the individual patient. The effects of disease are called symptoms,* therefore the definition of a disease may be expressed as *a symptom-group of determinate causation.*

But it is evident that with the diseases of parasitism, poisoning, and trauma, the afflictions of man are not exhausted. Incomplete development is the cause of many well-known congenital diseases. Again, children born in apparent good health and kept under the best hygienic conditions yet suffer from diabetes, asthma, epilepsy, and other serious diseases. The cause in these cases is a constitutional defect, received nearly always through the channel of heredity. Middle-aged and old people break down in many and quite definite ways as a result of unphysiological stress (overwork), lack of physiological strain (deficient work), or as a result of wear and tear. Thus we see that from childhood to old age man suffers from diseases that are not due to causes arising in his environment; they are therefore due to causes arising in his constitution, to causes of intrinsic as distinguished from those of extrinsic origin. And so we arrive at a description that is applicable to every known disease, and that will furnish a standard by which to judge of the genuineness of diseases yet to be discovered. When a recurrent, recognisable symptom-group has been correlated with one of the causes of disease mentioned a disease has been identified, but nothing less will satisfy the demands of medical science: and as regards diagnosis, when effect (symptom) has been traced to cause in an individual a diagnosis has been made, but nothing less will answer; no naming of symptoms apart from their cause can complete the scientific problem that every case sets us.

Definition and classification are interdependent; if we can describe the members of any series of natural processes accurately they will take their places in an orderly record, and this record will, in fact, be a scientific, a natural classification. On the other hand, failing accurate description of the processes, classification must be done on some artificial system. All diseases have a common origin in natural, knowable causation; this is the basis of the natural classification. There are two primary divisions; one contains all diseases due to causes of extrinsic, the other those due to causes of intrinsic, origin. The first is divided into groups corresponding with the causes mentioned—parasitism, poisoning, and traumatism; each group would fall into sub-groups that do not concern us here. The second primary division falls similarly into groups and subgroups under the intrinsic causes of disease—incomplete development, constitutional defects, overwork, deficient work, and wear and tear. In order to bring the scheme vividly and concisely before the reader's mind, I shall set down in the shape of a chart the headings of the main groups into which all diseases

* The word "symptoms" is used in this Paper to include both "signs" and "symptoms."

would divide themselves if described and classified on the basis of causation. I am well aware of the intricate questions that must arise when one comes to deal with the details and fine distinctions of classification; there are problems arising from the merging of one group into another, from the co-existence of two or more diseases in the individual, from the working of two or more causes in similar directions, and so on; but these questions, important as they are, do not affect the main principles, and are best left on one side till the principles are settled.

Names such as gout and diabetes occur here that have been elsewhere characterised as spurious diseases. Here the symptoms have been correlated with their causes, and so constitute true diseases. In the present-day text-books the same symptoms stand alone for diseases, although of indeterminate causation. Thus under gout and diabetes there is at present no attempt to differentiate the extrinsically from the intrinsically caused disease, with the result that these symptom-groups are at present spurious diseases.

Intrinsic Causes.

| Incomplete Development | Constitutional Defects | Over-work. | Deficient Work. | Wear and Tear. |
|---|---|---|---|---|
| Spina bifida<br>Idiocy<br>Congenital heart defects | Gout<br>Asthma<br>Diabetes<br>Hysteria<br>Neurasthenia | Various symptoms of breakdown in nervous and other systems | Obesity<br>Dyspepsia<br>Invalidism | Old age changes leading to deafness, blindness, arterio-sclerosis and its consequences |

Extrinsic Causes.

| Parasitism. | Poisoning. | Traumatism. |
|---|---|---|
| Tuberculosis<br>Malaria<br>Hydatid disease<br>Scabies | Lead poisoning<br>Rickets<br>Scurvy<br>Alcoholism | Heat stroke<br>Frost bite<br>Housemaid's knee<br>Fractures<br>Wounds |

The above chart shows clearly the meaning of what has been said of the definition and classification of diseases. Roughly, those set down in this chart represent all the causes that give rise to disease in man, and having once arrived at the true description of a disease as a symptom-group of determinate causation, it is evident that every disease must find its place in one of these groups. Symptoms of disease never arise in man haphazard, nor in the absence of some discoverable cause, therefore, however difficult the correlation of cause and effect may be, it is never impossible, and when we cannot succeed it is our duty as scientific men to recognise candidly that failure is due to our lack of capacity and not to the absence of a cause. It is often said that the cause of a disease is as indefinable and as elusive as the disease itself, or, what is much the same thing, that no disease is due to a single cause. This difficulty may be dealt with here parenthetically. It is maintained, for instance, that exhaustion, exposure, and consequent lowered vitality are predisposing causes of pneumonia, while the parasitism of the pneumococcus is the exciting cause; so that even in the case of such a definite disease it is impossible to assign a single cause. The supposed difficulty arises from a misuse of terms, and is a survival from the time when there was hardly any real knowledge of the causation of disease, and when medical science was consequently in its infancy. There is only one possible cause of pneumonia; no case ever arose in the absence of the specific microbe; all other so-called causes are merely predisposing conditions that led to the atack in some individual case.

The case of intrinsically caused diseases is precisely similar; for instance, under stress we meet with hysterical symptoms in people who are undoubtedly possessed of normal mental balance, but these cases are not examples of true hysteria, of the disease hysteria. Before diagnosing hysteria in a patient it is our business to determine, taking the circumstances that led to the attack into consideration, whether we are justified in concluding that the patient is not possessed of normal self-control. The cause of hysteria is intrinsic; it is a constitutional defect that may be described as a lack of mental stability. In the absence of this cause no case of hysteria, properly so-called, occurs any more than does a case of pneumonia in the absence of the pneumococcus. There is always this double problem to be dealt with: we must make a diagnosis, we must trace effect to cause; and we must also determine the particular conditions that led to the attack under consideration, but the two must mentally be kept apart. If it were agreed that we do know what is a disease, and that every cause of disease, whether intrinsic or extrinsic in origin, is comprehensible, single, and discoverable, the science of medicine would be easily put in order so that the line might be definitely drawn between what we know and what we do not know: diagnosis would then have a definite and fixed value; it would mean the tracing of effect to cause in the individual patient, nothing more and nothing less.

It will be noticed that this chart corresponds to a certain extent with the present method of classification; the diseases of parasitism and of poisoning, and to a certain extent those of traumatism, correspond with, for instance, the "Nomenclature of Diseases of the College of Physicians." The introduction of diseases of intrinsic causation is the most obvious change; about the necessity for this there can hardly be any serious difference. I can with difficulty imagine anyone maintaining that it is scientifically sound to class together, as suffering from the same specific disease, all those who show diabetic symptoms, no matter what the cause of the symptoms may be. It is not a case for argument but for reflection; if a disease—as is already recognised in the case of typhoid fever, lead poisoning, or heat-stroke—represents cause and effect, then diabetes is clearly not properly described as a disease; the profession is at present halting between two opinions. Diabetic symptoms caused by a constitutional defect must be recognised as a true disease; here cause and effect are correlated, but a certain symptom-group labelled diabetes, without reference to cause, is an example of the spurious disease of the symptom-group of indeterminate causation. Asthma, epilepsy, chorea, and gout are other examples of this very numerous class.

A further fundamental alteration in the aspect of our science that the adoption of the method here advocated would bring about must now be described. I refer to the exclusion from such a volume as the Nomenclature of the College of all spurious diseases. The so-called diseases of morbid anatomy stand first in importance. The dogma on which all Virchow's work as a physician rests is the assertion that every chronic disease is rooted in an organ. Virchow is immortal as an anatomist, but when he attempted to make his special branch of anatomy take the place of the science of medicine he was as hopelessly in error as the humoral pathologists had been whose rusty speculations he swept away with so much scorn. It is just as plain to-day that Virchow was wrong in thinking he had found the cause of a disease in the morbid anatomy of an organ as it was plain to him that the humoralist was on a hopeless quest in looking for the cause of a disease in the false mixings of the blood. We all know nowadays, though we do not all care to realise it, that in speaking of Bright's disease, Hodgkin's disease, Paget's disease, infantile paralysis, tabes, myxœdema, status lymphaticus, and so on in ever-increasing variety, we are naming spurious diseases, merely labelled symptom-groups. The question of diagnosis is equally clear. When we diagnose typhoid fever or lead poisoning we commit ourselves to a definite opinion, to a correlation of cause and effect; whereas in the case of infantile paralysis or of any other "disease" of morbid anatomy we commit ourselves to nothing more than the labelling of an easily recognised symptom-group, we are content with a wholly illusory diagnosis. However sacred his post-mortem lesions may be to the pathologist, they remain but symptoms; they are no doubt more "real" than the humours and fluxes of the humoralist, but they are not one whit more the causes of a disease. Some may say that I am beating a straw man of my own making, that it is clearly understood that in speaking of or in "diagnosing" his "organic diseases" the pathologist does not profess to be dealing with true diseases. My impression is that when a book is written on the diseases of the liver, of the heart, of the lungs, of the nervous system, of the brain, of the skin, or of the mind, the writer really does intend to use the word disease in its particular positive sense, and that therefore he is misusing it to the confusion of himself and of the reader. My contention is that the time has arrived for the profession to decide whether we can with impunity admit any other use of the word disease than the two that have been specified, and, if so, what the other meaning is.

For the same reason, that they do not represent cause and effect, all "local diseases" are spurious. No disturbance of the skin, however severe, can by any stretch of the imagination be looked upon as a disease if a disease is to represent cause and effect. Necrosing ethmoiditis and atrophic rhinitis are clearly only formidable names given to symptoms, not because they have been traced to their cause, but in order that they may be raised to the status of a disease, and so become objects of "special diagnosis." Glaucoma and cataract are in a similar position; when they have been named by a specialist they have been "diagnosed"; but the problem that has been solved when typhoid fever, lead-poisoning, or any other true disease has been diagnosed has clearly not been solved when a "disease" of the nose or eye has been named. It is unnecessary to multiply examples, and every medical man has ample material in his own experience to convince him on reflection that there is no difficulty whatever in concluding in the case of any so-called disease of whatever variety, whether it is true or false according to the standard here set up.

I have been asked over and over again what I propose to do with all these spurious diseases when excluded from the category of true diseases. The answer is obvious; they should be excluded in order that every practitioner—regardless of caste—may realise that in naming them he had only attempted an illusory diagnosis, that he had not traced effect to cause, and that his scientific work is consequently left deliberately incomplete. This separation of true from false would enable the whole profession to realise that until every symptom of disease has been correlated with the cause, or causes, from which it arises there is still purely scientific work for us all in our daily routine. So long as the student is taught to "diagnose" infantile paralysis, Hebra's prurigo, myxœdema, diabetes, glaucoma, cancer, mycosis fungoides, and so on without looking for the cause in every case, so long will the body of the profession be more than half paralysed for the search after knowledge, and so long will ignorance be cloaked and stereotyped.

The question here raised is apt to take on a highly controversial quality if the discussion wanders to the practical results that might ensue if the method now advocated were adopted. I shall therefore end as I began, by insisting that what has been written is a plain statement on a purely scientific subject; it is susceptible either of substantiation or refutation, and to this end I invite criticism. It has been stated, I hope intelligibly, that the pure science of medicine is not abreast of the other branches of science, that it is not pursued in accordance with modern method. The failure to recognise the invariable, natural sequence of cause and effect in the description and classification of diseases is to my mind a gross and palpable error that vitiates all our scientific method. Surely the indefinable and unclassifiable diseases of the college classification belong properly to the same period as the specially created species of pre-Darwinian natural history.

The profession is at a parting of the ways; the path it is on is leading to a greater and greater dispersion of scientific effort, to a more and more minute specialisation in the study of symptoms, and so to an attempted subdivision of our science, of which no one can see the end, nor even the limit. The path that I have tried to point out would lead on the other hand to the immediate unification of our science, and to the establishment once for all of the complete clinical correlation of cause and effect over the whole territory of human disease as the true scientific goal towards which the whole profession must strive.

